# A Framework for Detecting Thyroid Cancer from Ultrasound and Histopathological Images Using Deep Learning, Meta-Heuristics, and MCDM Algorithms

**DOI:** 10.3390/jimaging9090173

**Published:** 2023-08-27

**Authors:** Rohit Sharma, Gautam Kumar Mahanti, Ganapati Panda, Adyasha Rath, Sujata Dash, Saurav Mallik, Ruifeng Hu

**Affiliations:** 1 Department of Electronics and Communication Engineering, National Institute of Technology, Durgapur 713209, India; rohithmr.21791@gmail.com (R.S.); gautammahanti@yahoo.com (G.K.M.); 2 Department of Electronics and Communication Engineering, C.V. Raman Global University, Bhubaneswar 752054, India; ganapati.panda@gmail.com; 3 Department of Computer Science and Engineering, C.V. Raman Global University, Bhubaneswar 752054, India; adyasha.rath@cgu-odisha.ac.in; 4 Department of Information Technology, Nagaland University, Dimapur 797112, India; sujata238dash@gmail.com; 5 Department of Environmental Health, Harvard T H Chan School of Public Health, Boston, MA 02115, USA; 6 Department of Pharmacology & Toxicology, The University of Arizona, Tucson, MA 85721, USA; 7 Department of Neurology, Brigham and Women’s Hospital, Harvard Medical School, Boston, MA 02115, USA

**Keywords:** dimensionality reduction, image transformers, method based on the removal effects of criteria, Mixer model, technique for order of preference by similarity to ideal solution, transfer learning, weighted average ensemble learning

## Abstract

Computer-assisted diagnostic systems have been developed to aid doctors in diagnosing thyroid-related abnormalities. The aim of this research is to improve the diagnosis accuracy of thyroid abnormality detection models that can be utilized to alleviate undue pressure on healthcare professionals. In this research, we proposed deep learning, metaheuristics, and a MCDM algorithms-based framework to detect thyroid-related abnormalities from ultrasound and histopathological images. The proposed method uses three recently developed deep learning techniques (DeiT, Swin Transformer, and Mixer-MLP) to extract features from the thyroid image datasets. The feature extraction techniques are based on the Image Transformer and MLP models. There is a large number of redundant features that can overfit the classifiers and reduce the generalization capabilities of the classifiers. In order to avoid the overfitting problem, six feature transformation techniques (PCA, TSVD, FastICA, ISOMAP, LLE, and UMP) are analyzed to reduce the dimensionality of the data. There are five different classifiers (LR, NB, SVC, KNN, and RF) evaluated using the 5-fold stratified cross-validation technique on the transformed dataset. Both datasets exhibit large class imbalances and hence, the stratified cross-validation technique is used to evaluate the performance. The MEREC-TOPSIS MCDM technique is used for ranking the evaluated models at different analysis stages. In the first stage, the best feature extraction and classification techniques are chosen, whereas, in the second stage, the best dimensionality reduction method is evaluated in wrapper feature selection mode. Two best-ranked models are further selected for the weighted average ensemble learning and features selection using the recently proposed meta-heuristics FOX-optimization algorithm. The PCA+FOX optimization-based feature selection + random forest model achieved the highest TOPSIS score and performed exceptionally well with an accuracy of 99.13%, F2-score of 98.82%, and AUC-ROC score of 99.13% on the ultrasound dataset. Similarly, the model achieved an accuracy score of 90.65%, an F2-score of 92.01%, and an AUC-ROC score of 95.48% on the histopathological dataset. This study exploits the combination novelty of different algorithms in order to improve the thyroid cancer diagnosis capabilities. This proposed framework outperforms the current state-of-the-art diagnostic methods for thyroid-related abnormalities in ultrasound and histopathological datasets and can significantly aid medical professionals by reducing the excessive burden on the medical fraternity.

## 1. Introduction

Thyroid cancer is a type of cancer that affects the thyroid gland, a small butterfly-shaped gland located in the neck. The thyroid gland produces hormones that regulate metabolism, heart rate, and body temperature. With early diagnosis and appropriate treatment, the prognosis for thyroid cancer is generally good. Medical experts, including doctors, nurses, and other healthcare professionals, are essential for providing healthcare services to the population. They are responsible for ensuring their patients’ well-being, including diagnosing and treating illnesses, providing emotional support, and making difficult decisions. This burden is amplified in times of crisis, such as pandemics or natural disasters, where the healthcare system may become overwhelmed, and medical professionals may have to work extended hours or in difficult conditions. Machine learning-based models have shown promising results in reducing the burden on healthcare experts. These models can be trained to analyze vast amounts of medical data and make predictions or provide insights to assist medical professionals in diagnosis, treatment, and care [1].

The accuracy of thyroid computer-aided diagnosis (CAD) systems is of the utmost significance in healthcare, as it directly impacts the diagnosis and treatment of patients. Inaccurate CAD models may result in missed or false-positive diagnoses, leading to delays in treatment or unnecessary interventions [2].

### 1.1. Background

Various imaging techniques can be used to diagnose thyroid cancer. Some of the most commonly used imaging techniques include ultrasound, computed tomography (CT) scans, magnetic resonance imaging (MRI), radioactive iodine scanning, and histopathological imaging [3,4].

Many researchers have proposed artificial intelligence-based CAD models for thyroid abnormality detection using ultrasound and histopathological images. In [5], Xu et al. examined a diagnostic model that utilized contrast-enhanced thyroid ultrasound images. The model used a convolutional neural network (CNN) as a feature extractor and a long short-term memory (LSTM) as a classifier. Zhao et al. [6] proposed a method that combines image texture features with CNN-extracted features for thyroid classification using ultrasound images. Rehman et al. [7] utilized the U-Net model to segment thyroid ultrasound images. The authors in [8] used CNN to classify thyroid and breast cancer ultrasound images. Liu et al. [9] introduced a multi-scale region-based detection network that used Resnet50 as the backbone network and ZFnet as a classifier. The proposed network achieved an improvement in accuracy of 0.90%. In [10], Chi et al. employed an inception network-based model for thyroid nodule classification and used transfer learning to mitigate overfitting, achieving an accuracy of 79.36%. Likewise, in [11], researchers used transfer learning-based VGG16 and GoogLeNet models, with achieved accuracies of 77.57% and 79.36%, respectively. Nguyen et al. [12,13] utilized the publicly available TDID dataset, incorporating knowledge of both spatial and frequency domains. The frequency-domain FFT is used to classify easy samples into three categories: malignant, benign, and ambiguous. A spatial domain model was used to categorize ambiguous samples. In [13], Nguyen et al. utilized the same technique as [12] but used a weighted binary cross-entropy loss function to address the class imbalance issue. In [12,13], they achieved 90.88% and 92.05% classification accuracies, respectively. Additionally, the authors employed the voting ensemble method with base CNN models. Sharma et al. [14] trained deep vision Transformer and Mixer models in weighted average ensemble learning configuration. They used the hunger games search algorithm to obtain the ensemble weights and the D-CRITIC TOPSIS method was utilized for ranking the models. The best model achieved classification accuracies of 82.17% and 89.70% for 70:30 and 80:20 split cases. Sun et al. proposed the TC-ViT model, which consists of a vision transformer with contrast learning for thyroid cancer detection. The researchers collected a private dataset of 794 thyroid images, where samples below the TI-RADS score of 4 are marked as benign, whereas the images with a TI-RADS score equal to and above 4 were treated as benign or cancerous.

Recent studies showed the promising application of AI tools in histo-cytology for standardization and enhancing the accuracy of indeterminate thyroid nodule classification [15,16]. The authors used digital images obtained from the thyroid fine needle aspiration biopsy technique. The study performed by Hirokawa et al. in [17] demonstrated the EfficientNetV2-L image classification model for the thyroid fine needle aspiration cytology. Similarly, Kezalarian [18] explored the application of AI to distinguish follicular carcinoma from follicular adenoma, whereas Alabrak et al. [19] proposed a CNN model to classify the same kind of problem and achieved an accuracy of 78%, a sensitivity of 88.40%, a specificity of 64% and AUC-score value of 0.87. Girolami et al. [20] presented a review article on automatic whole slide image analysis for thyroid pathology using AI tools. The study utilized a modified QUADAS-2 tool for the analysis of whole slide images. According to Wang et al. [21], they gathered 11,715 unique histopathological images from 806 patients. They trained VGG-19 and Inception-ResNet-v2 models on these images and attained accuracies of 97.34% and 94.42%, respectively. These models were utilized to categorize seven different types of thyroid abnormalities. In their study, Chandio et al. [22] suggested a decision support system for detecting medullary thyroid cancer (MTC) using a convolutional neural network (CNN). The authors trained the models on cytological images and obtained an accuracy of 99.00%. Hossiny et al. [23] employed cascaded CNN and split classification techniques to categorize thyroid tumors into three types: follicular adenoma, follicular carcinoma, and papillary carcinoma. The achieved accuracy in their study is 98.74%. Do et al. [24] proposed a deep-learning model called MI Inception-v3 to detect thyroid cancer. The authors compared the proposed MI Inception-v3 model with the Inception-v3 model. They observed that the classification accuracy on Tharun and Thompson dataset increased from 85.73% to 89.87% using the MI Inception-v3 model. Similarly, on Nikiforov’s dataset, the accuracy improved from 72.65% to 74.47%. Bohland et al. [25] compared feature-based and deep learning-based classification models for thyroid gland tumor classification. The feature-based method comprises cell nucleus segmentation, feature extraction, and classification using different classifiers. On the other hand, the deep learning-based method employs a convolutional neural network that directly classifies input images without cell nucleus segmentation. The authors observed that on the Tharun and Thompson datasets, the feature-based classification achieved an accuracy of 89.70%, while the deep learning-based classification achieved 89.10%.

Recently, Transformer and Mixer models have effectively been utilized for vision tasks, and they have shown comparable results to CNN models. These deep-learning models can be trained from scratch, or pre-trained models can be employed for feature extraction [26,27,28]. However, there is no evidence to date on the usage of Transformer and Mixer models for thyroid image feature extraction.

The extracted feature vector obtained from the dataset contains redundant features, which can result in over-fitting of the classifier model. To address this issue, various dimensionality reduction techniques can be employed to transform high-dimensional data into a relevant lower-dimensional space. Espadoto et al. [29] conducted a comprehensive quantitative survey of such methods. The researchers created a benchmark that consists of 44 techniques, which include different combinations of their parameter values, 18 datasets, and 7 quality metrics, and the results of their study are fairly impressive.

Meta-heuristic-based feature selection techniques have gained attention in recent years for handling high-dimensional datasets. A survey conducted by Yab et al. [30] found that moth flame optimization performs well in filter-based methods, while the cuckoo optimization algorithm works well in wrapper-based methods. The whale optimization algorithm was found to perform well in both scenarios. Regarding classifier preferences, the filter-based method prefers SVM, DT, and NB, while the wrapper-based method prefers KNN.

In the medical domain, datasets are generally tiny, and it can be difficult to increase the size of the dataset due to limited patient participation, privacy, and costly testing modalities. One way to enhance the accuracy of classification models is by using an ensemble of multiple weak learners [31]. The weighted average ensemble approach produced encouraging results, but finding the optimal weights is a challenging optimization task [32]. Recently, a new optimization algorithm called FOX optimization was developed, outperforming other meta-heuristic algorithms in traditional benchmarks. This algorithm utilizes techniques for measuring the distance between a fox and its prey to make efficient jumps. However, this algorithm has yet to be applied to feature selection or weighted average ensemble learning [33].

It is essential to evaluate and compare the proposed models using benchmarking techniques. However, it is difficult to select the best model due to conflicting performance results. To address this issue, multi-criteria decision-making (MCDM) methods have successfully been used to rank the models based on various performance metrics. In a study by Mohammed et al. [34], a TOPSIS benchmark was proposed for ranking machine learning models for COVID-19 diagnosis. The weights assigned to each criterion significantly impact the ranking process. Nguyen et al. [35] analyzed and compared the recently proposed MEREC weighting technique with other studies, but there is no evidence of MEREC-TOPSIS being used for ranking and selecting the best models in the literature.

### 1.2. Research Gap

From the literature survey, the following is evident:

- Transformer and Mixer models have not yet explored for thyroid image feature extraction. Most studies took place using CNN models.
- The dimensionality reduction techniques have not been analyzed on extracted features from Transformer and Mixer models.
- The FOX optimization algorithm has neither been applied for feature selection nor for weighted average ensemble learning.
- MEREC weighting with the TOPSIS method has not been evaluated for thyroid cancer application.
- The performance of the CAD model for thyroid cancer diagnosis needs to be improved for much better results.
- The proposed model needs to be evaluated on imbalanced thyroid datasets using a stratified sampling technique for efficient class representation.

### 1.3. Our Contribution

- This work uses three deep learning-based feature extraction techniques (Deit, Swin Transformer, and Mixer-MLP) to extract feature vectors from histopathological and ultrasound images.
- Six feature reductions (PCA, TSVD, FastICA, ISOMAP, LLE, and UMAP) techniques are used to reduce the dimensionality of the extracted feature space.
- The MEREC-TOPSIS technique ranks and selects the best-evaluated models at different stages.
- The recently invented FOX optimization algorithm is used for feature selection and weighted average ensemble learning.
- The ensemble and feature selection-based models are ranked at the last stage, and the best model is compared with the state-of-the-art techniques.
  The proposed framework demonstrates the analysis as well as the applicability of the Transformer, Mixer, dimensionality reduction, feature selection, FOX optimization, and MEREC-TOPSIS techniques in thyroid cancer detection. The proposed framework also explores the weighted average ensemble using the FOX optimization algorithm, and a comparative study is also shown in this study. The framework showed combinational novelty in the process and outperformed the existing techniques. According to the review of the existing literature, this technique is novel and has not been employed by any researchers previously for detecting thyroid cancer.

## 2. Materials, Methods and Theoretical Overview

### 2.1. Materials

The proposed framework is evaluated on two different image datasets: ultrasound and histopathological datasets.

- Ultrasound dataset: While several studies have investigated ultrasound imaging for thyroid cancer diagnosis, most data sources used in these studies are not publicly available. Gathering a significant amount of data is challenging due to time constraints, the precise nature of medical modalities, the need for patient involvement, and the cost of image collection equipment. The Thyroid Digital Image Database (TDID) was used in this study to address these difficulties. The TDID dataset, collected by Pedraza et al. [36] and published by the Universidad Nacional de Colombia in 2015, is publicly available. The dataset consists of 298 patients involved in the data collection process. The dataset has previously been used in research to address thyroid nodule classification challenges, and it contains TIRAD scores and nodule localization details for each patient, with one or more samples taken from each patient. Each ultrasound image in the dataset is 560 × 360 pixels in size. The TI-RADS score indicates the health of the thyroid nodule and can range from 1 to 5. Scores 1, 2, and 3 indicate benign thyroid nodules, while scores 4a, 4b, 4c, and 5 show malignant thyroid nodules. The images which contain benign thyroid nodules are treated as cancerous images, whereas the images with malignant nodules are treated as non-cancerous thyroid samples. The dataset is used for binary class classification problems. There are a total of 347 thyroid nodule sample images retrieved from 298 patients. Out of 347 images, 286 images contain thyroid nodules with TI-RADS scores less than 4 and are considered non-cancerous (benign) thyroid cases. The remaining 61 images have TI-RADS scores greater than or equal to 4 and are treated as cancerous (malignant) samples. The dataset is highly imbalanced, and hence stratified oversampling is proposed to evaluate the performance.
- Histopathological Dataset: For histopathological thyroid images, the dataset is provided by Thompson et al. on request. The dataset developed by Tharun and Thompson [37] includes a group of 156 thyroid gland tumors obtained from the pathology archives at the University Clinic Schleswig-Holstein, Campus Luebeck (138 tumors) and the Woodland Hills Medical Center, Woodland Hills, California (18 tumors). A single hematoxylin and eosin-stained section was selected from each tumor and scanned using the Ventana iScan HT (Roche Diagnostics, Basel, Switzerland). The whole slide images were captured at 40× magnification with a resolution of 0.23 μm/px and processed as 8-bit color depth RGB images. Two pathologists independently classified each whole slide image, and any confusion was resolved through discussion to reach a consensus for each case. The dataset comprised five distinct entities: follicular thyroid carcinoma (FTC) with 32 patients, follicular thyroid adenoma (FA) with 53 patients, noninvasive follicular thyroid neoplasm with papillary-like nuclear features (NIFTP) with 9 patients, follicular variant papillary thyroid carcinoma (FVPTC) with 9 patients, and classical papillary thyroid carcinoma (PTC) with 53 patients. Oncocytic neoplasms, which can be easily classified based on cytoplasmic and architectural features, were excluded from the dataset. To facilitate the experiments and make the problem a binary classification task, the five different entities were combined into two groups: non-PTC-like (FTC, FA, 85 patients) and PTC-like (NIFTP, FVPTC, and PTC, 71 patients). From each whole slide image, a pathologist extracted representative images from the neoplastic areas. In 147 out of 156 entities, ten non-overlapping images of size 1916 × 1053 px were extracted from the neoplastic areas. However, only one to six images were available for the remaining nine cases with small neoplasm areas.

### 2.2. Methodology

**Figure 1:**
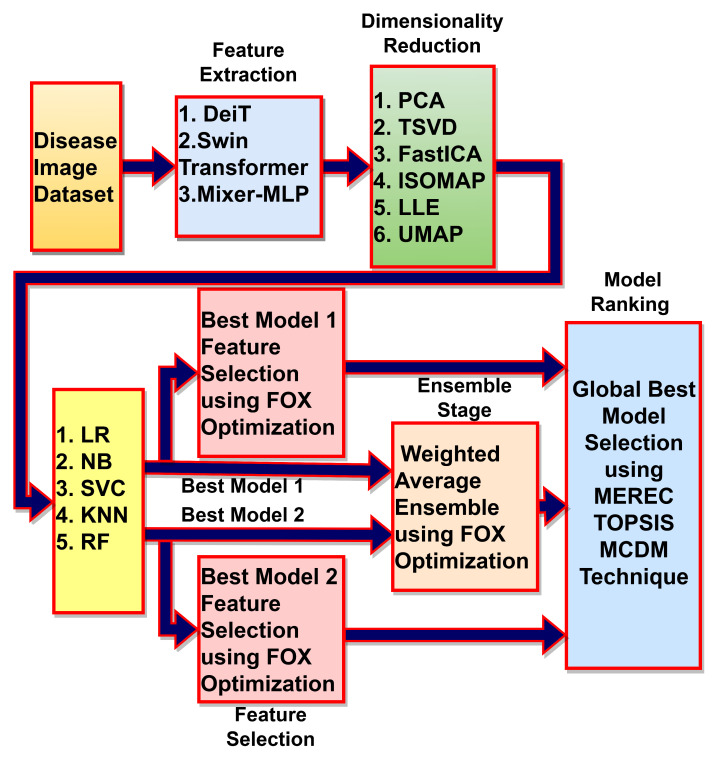
Deep learning, meta-heuristics and MCDM algorithms-based framework for thyroid abnormality detection.

The thyroid abnormality detection framework is depicted in Figure 1, which provides an overview of the different blocks used in the proposed framework. These blocks are discussed in subsequent subsections to provide a comprehensive understanding of the proposed approach.

#### 2.2.1. Dataset Cleaning

The ultrasound dataset is 560 × 360 pixels in size and contains black regions and markers. This black background region and markers have no significant information, which can add redundancy and bias to the classification models. These regions and markers are removed using the thresholding technique proposed by Nguyen et al. [12,13]. The images are resized into 224 × 224 pixels. For the Tharun Thompson dataset, the size of the image is large. Each image is divided into 45 patches with a horizontal and vertical shifting window of size 224 × 224. The data augmentation technique is used to enlarge the image to obtain an integer multiple of window size.

#### 2.2.2. Feature Extraction

Pre-trained models of three deep learning-based feature extraction techniques (DeiT, Swin Transformer and Mixer-MLP) are used for the feature selection. For ultrasound images, the extracted feature vector is directly used for further machine learning pipeline, whereas for the histopathological dataset, 45 patch images provide the same number of feature vectors. These feature vectors are fused together for further pre-processing.

#### 2.2.3. Feature Reduction

Six dimensionality reduction techniques (PCA, SVD, FASTICA, ISOMAP, LLE and UMAP) are used to transform the higher-dimension features into lower-dimension space.

#### 2.2.4. Feature Selection

The lower-dimension features are further given to the feature selection block to reduce redundant features. The FOX optimization algorithm selects the best features, using accuracy as the cost function. The task is treated as a minimization problem.

#### 2.2.5. Weighted Average Ensemble

The two best models are selected after the feature reduction stage, and the weighted ensemble is performed, where weights are optimized using the FOX optimization technique with accuracy as the cost function.

#### 2.2.6. Optimal Model Selection

Different combinations of feature selection, feature reduction and ensemble learning are tried. The MEREC-TOPSIS method is used to benchmark the models based on three criteria (Accuracy, F2-score, and AUC-ROC score) for every possible combination. The models are ranked after every stage, and the best strategies are forwarded to the next stage.

### 2.3. Theoretical Overview

#### 2.3.1. Feature Extraction Techniques

- Data-efficient image Transformer: The data-efficient Image Transformer (DeiT) was proposed by Touvron et al. [28] and comprises three main components, namely knowledge distillation, regularization, and augmentation. The model involves two models used for training, the teacher model (pre-trained RegNetY-16GF) and the student model (Vision Transformer). Initially, the student network is trained on the dataset, and the cross-entropy loss is calculated. The knowledge distillation component is critical in the DeiT model, wherein a pre-trained model calculates the output probabilities for different classes with the soft-max of a specific temperature parameter. These probabilities are compared with the ground truth, and the distillation loss is calculated. The cross-entropy and distillation losses are added together, and the overall estimated loss function is used to train the student model. Many versions of DeiT models are available, and this study employed the DeiT-Small model with 22 million parameters.
- Swin Transformer: According to Liu et al. [27], the Swin Transformer is an architecture that builds upon the Vision Image Transformer (ViT), but instead of a uniform patch size, it uses a hierarchical patch structure. The Swin Transformer comprises four key components: patch partitioning, linear embedding, Swin Transformer block, and patch merging layer. For feature extraction in our work, a pre-trained Swin Transformer (Swin-S version) is utilized, which has 50 million parameters and a linear projection dimension of 96. In the Swin Transformer, the Transformer layer uses limited attention and replaces the standard multi-head attention with shifted-window multi-head attention (Shifted-MSA). The patch merger layer is utilized to merge neighboring patches. Compared to ViT, the Swin Transformer is better at capturing detailed image descriptions.
- Mixer-MLP: Tolstikhin and colleagues [26] introduced Mixer-MLP for vision tasks. This straightforward design is founded on a multi-layer perceptron. The picture is partitioned into patches and projected into linear embeddings, also known as tokens. Two types of MLP layers are present. The channel mixing layer operates on each token independently, while the token mixing layer enables communication among all the channels. Before classification, the global pooling layer and skip connections are utilized at the output. In this research, the pre-trained B-16 version of mixer-MLP is employed.

#### 2.3.2. Dimensionality Reduction Techniques

There are different feature transformation techniques employed in this study [29].

- Principal component analysis: PCA is a mathematical method that converts multiple correlated variables into a smaller group of uncorrelated variables known as principal components. This technique identifies linear combinations of variables that capture the most variation in the data. The derived principal components can be further analyzed or visualized.
- Truncated singular value decomposition: TSVD, or truncated singular value decomposition, is a method used to decompose a matrix into its singular values and corresponding vectors. Unlike the full SVD, TSVD only keeps the top singular values and vectors, allowing for more efficient computation and reduced noise and dimensionality. TSVD can also help with ill-conditioned matrices, where the full SVD may fail to converge.
- Fast independent component analysis: Fast independent component analysis (FastICA) is a popular independent component analysis (ICA) method. It is a computational technique that separates a multivariate signal into independent, non-Gaussian components. FastICA identifies the underlying sources of variability in the data by maximizing the independence between the extracted components. FastICA has the advantage of being fast, computationally efficient, and flexible.
- Isometric feature mapping: Isometric feature mapping (ISOMAP) is a nonlinear dimensionality reduction technique preserving the geodesic distances between the data points. It works by constructing a neighborhood graph based on the Euclidean distances between the data points and then approximating the geodesic distances on the manifold by finding the shortest path through this graph. The final embedding is obtained through classical multidimensional scaling (MDS) of the geodesic distances. ISOMAP has been shown to be effective in preserving the global structure of the data, especially in cases where the data lie on a low-dimensional nonlinear manifold embedded in a high-dimensional space.
- Locally linear embedding: Locally linear embedding (LLE) is a nonlinear technique that aims to reduce the dimensionality of data. The method calculates the local relationships between points and their neighbors and uses these relationships to construct a lower-dimensional representation of the data. It is useful for nonlinear manifolds, where linear techniques like PCA may not work well.
- Uniform manifold approximation and projection: Uniform manifold approximation and projection (UMAP) is a non-linear dimensionality reduction technique that constructs a high-dimensional graph of the data points and then optimizes a low-dimensional graph that preserves the topology and geometry of the high-dimensional graph. UMAP can handle complex and non-linear relationships between data points and is often faster and more scalable than other non-linear techniques, like t-SNE.

#### 2.3.3. Meta-Heuristic Algorithm for Feature Selection

- FOX optimization algorithm: This algorithm, proposed in 2022, imitates the behavior of a red fox when it is hunting its prey in the snow [33]. The algorithm consists of five main steps. First, the fox searches for prey randomly as the snow covers the ground. Then, it uses ultrasound to locate the prey and moves closer to it. Next, it determines the distance between itself and the prey by analyzing the sound and time difference. After that, it calculates the necessary jump to catch the prey. Finally, the algorithm performs random walking based on the minimum time and the best position to continue searching for prey. The optimization algorithm considers exploration and exploitation to reach the best global solution.

#### 2.3.4. MCDM Method for Ranking the Models

The MEREC-TOPSIS method is used for rank evaluation of the models proposed and analyzed in this study [35].

- Method based on the removal effects of criteria: The method based on the removal effects of criteria (MEREC) method is a new objective weighting method that uses the removal effects of criteria in the decision matrix to determine their importance. Unlike the other methods, MEREC focuses on an exclusion perspective and removal effects to determine the objective criteria weights instead of the inclusion perspective.
- Technique for order of preference by similarity to ideal solution: The technique for order of preference by similarity to ideal solution (TOPSIS) is a decision-making method that evaluates alternatives based on their proximity to an ideal solution and distance to a negative ideal solution. To use TOPSIS, one constructs a normalized decision matrix, weights it, calculates the ideal and negative ideal solutions, and computes the separation measures for each alternative. The technique ranks alternatives by their proximity to the ideal solution and the importance of each criterion. This method is frequently used in fields such as finance, engineering, and management to assist with decision making.

## 3. Simulation-Based Experimental Results

There are two types of thyroid disease images: ultrasound and histopathological images. The proposed framework is applied to both types of images. The pre-trained models DeiT-Small, Swin Transformer, and Mixer-MLP type B-16 are used for feature extraction, and the sizes of the feature vectors for ultrasound images are 384, 1025, and 768, respectively. For histopathological images, there are 45 patch images, and hence, the size of the feature vectors after feature fusion are 17,280, 46,125, and 34,560 for the DeiT, Swin Transformer, and Mixer-MLP models, respectively. All the models are pre-trained on the ImageNet-1K dataset. The extracted features are reduced to 200 and 1000 using PCA for ultrasound and histopathological datasets. These reduced features are given to five classifiers (LR, NB, SVC, KNN, and RF). The classifiers are trained using a 5-fold stratified cross-validation technique. A total of 15 models are trained for both datasets based on feature extraction, PCA, and classifiers.

The results are displayed in Table 1 and Table 2. The model selection, which incorporates the optimal feature extraction and classifier, is performed using MEREC-TOPSIS. The assigned MEREC weights are provided in Table 3. From the results presented in the tables, it is evident that Model10 achieves the highest ranking for both datasets. Model10 utilizes the Swin Transformer as the feature extractor, PCA as the feature reducer, and random forest as the classifier.

In the next stage, the Swin Transformer and random forest are utilized to evaluate the performance of the dimensionality reduction techniques. The results are shown in Table 4 and Table 5, whereas the MEREC weights are displayed in Table 6, used for the calculation of the TOPSIS scores for both datasets.

After evaluating the TOPSIS scores, the two most favorable models were chosen. In the case of the ultrasound dataset, Model10 (which employs Swin Transformer, PCA, and random forest) is positioned at the top rank, while Model18 (which uses Swin Transformer, ISOMAP, and random forest) is in second place. Regarding the histopathological dataset, Model10, which utilizes Swin Transformer, PCA, and random forest, achieved the highest ranking, while Model19, employing Swin Transformer, LLE, and random forest, secured the second position.

These two best-selected models are then given to feature selection based on the FOX optimization algorithm. Also, these two models are used for weighted average ensemble, where weights are optimized using the FOX optimization algorithm. The results are shown in Table 7 and Table 8 for both datasets.

The MEREC weights for TOPSIS scores calculation for the performance metrics in Table 8 and Table 9 are calculated and given in Table 9.

**Figure 2:**
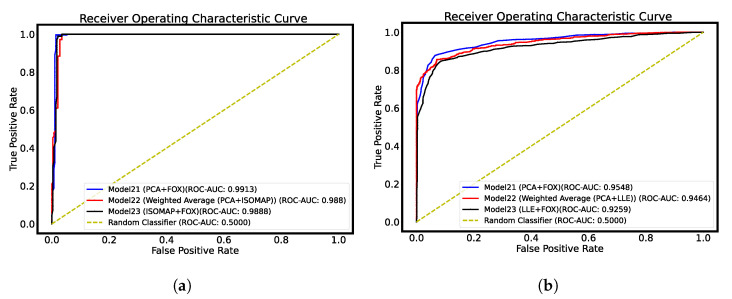
AUC-ROC curve plots for thyroid datasets. (**a**) Ultrasound dataset AUC-ROC plot; (**b**) histopathological dataset AUC-ROC plot.

**Figure 3:**
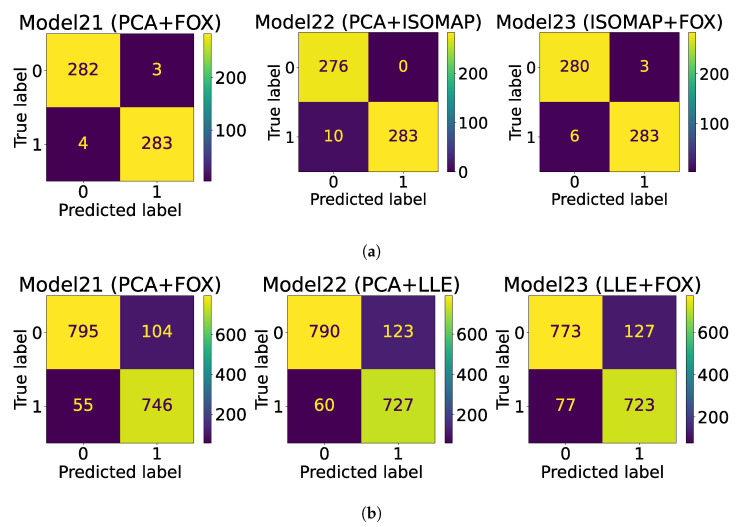
Confusion matrix plots. (**a**) Ultrasound dataset confusion matrix plot; (**b**) Histopathological dataset confusion matrix plot.

The AUC-ROC curves are plotted in Figure 2, whereas the confusion matrix is plotted in Figure 3 for both datasets.

**Figure 4:**
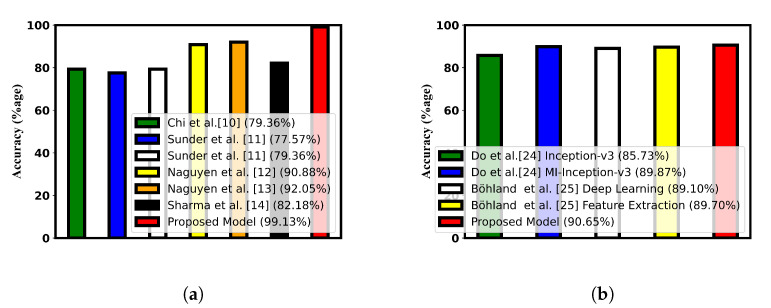
Accuracy comparison with the state-of-the art research. (**a**) Accuracy comparison for ultrasound dataset [10,11,12,13,14]; (**b**) accuracy comparison for histopathological dataset [24,25].

The accuracy compared with the state-of-the-art techniques is shown in Figure 4 with the help of bar charts. Table 10 and Table 11 show the comparison of different performance metrics with the current research.

## 4. Discussion

Based on the simulated experimental results, it is evident that model21, a combination model that utilizes Swin Transformer, PCA, and FOX optimization for feature selection, is the most effective model. This model was able to achieve the highest levels of accuracy on both the ultrasound and histopathological datasets, with accuracy rates of 99.13% and 90.65%, respectively. In comparison to other models, Model21 was able to achieve an improved accuracy of 6.08% on the ultrasound dataset [13] and 0.95% on the histopathological dataset [25]. The proposed model was successful in achieving improved recall and specificity values. In addition, the ensembled model (Model22) also demonstrated better performance compared to existing state-of-the-art techniques. However, it was not able to surpass the performance of Model21. The AUC score of 0.9545 obtained for the histopathological dataset represents a significant improvement, as it is higher than the score obtained by the feature extraction-based model proposed in the study by Bohland et al. [25]. In the confusion matrix plot, 0 corresponds to benign thyroid nodules, and 1 corresponds to malignant thyroid nodules for ultrasound-based thyroid cancer detection. On the other hand, for the Tharun Thomson dataset, the value of 0 represents non-papillary thyroid carcinoma, and the value of 1 represents papillary thyroid carcinoma.

## 5. Conclusions

Within this segment, we summarize the findings, limitations and future possibilities of the proposed research.

### 5.1. Findings

Based on the simulation results in this study, we discover the following:

- For both datasets, the RF classifier achieved the best performance results when combined with any of the three feature extraction techniques. The LR has the worst performance parameters with DeiT as a feature extraction model for histopathological datasets, whereas the NB classifier with Swin Transformer provides the worst TOPSIS score value for the ultrasound dataset.
- Swin Transformer has the best feature extraction capabilities and is ranked as the best feature extractor among the three techniques employed in our study.
- The study showed that the PCA outperformed all other five dimensionality reduction techniques for both datasets, whereas UMAP obtained the poorest results.
- The research study also demonstrated the feature selection capabilities of the FOX optimization algorithm. The best model is based on Swin Transformer, PCA, and RF with FOX optimization for feature selection purposes for both datasets. For the histopathological dataset, the LLE dimensionality reduction technique also showed promising results closer to the best PCA-based model, whereas the ISOMAP has a close contest with the PCA for the ultrasound dataset.
- The proposed framework outperforms all the existing state-of-the-art performance results obtained on both datasets. The F-score comparison showed that the proposed framework can also deal with the class imbalance issue as compared to the available methods proposed in the literature. By achieving higher values of specificity, the proposed model effectively reduced the false positive rate, which can lead to a decrease in the cost of medical procedures and a reduction in mental pressure on patients.

### 5.2. Limitations

- Although the proposed framework used a generalized process and can be utilized on any image dataset, this article only focused on two thyroid image datasets. This is due to the unavailability of the publicly standard thyroid datasets. Most of the existing studies demonstrated the performance of their proposed models on private datasets.
- The thyroid datasets are heterogeneous in nature and obtained from external sources. However, they are generalized frameworks and can be utilized in disease detection from any medical image dataset.

### 5.3. Real Life Applicability

The proposed model outperformed the ensemble model and only consists of a small model version of Swin Transformer for transfer learning, PCA for feature transformation, and random forest for classification. The proposed model has good performance results. This small model can be deployed on edge devices because they have limited computation power and energy resources. This model can be utilized for remote healthcare, where remote health centers provide patient samples. The extracted feature can then be transferred to a cloud-based random forest classifier to diagnose thyroid cancer. The thyroid diagnosis process can be integrated with emergency services, financial institutions, and electronic health record systems so that detecting thyroid cancer automatically triggers this integrated system. Medical data are generally susceptible, and transferring these data over the internet may sometimes pose privacy concerns. In such a case, the classifier can be trained in a federated learning mode, where the weights of the classifier can be used to train the global model deployed on the cloud. The transfer learning and single classifier make this model very easily deployable on edge devices. Reduced features using PCA can reduce the channel bandwidth required for data transfer over the wireless channel.

### 5.4. Future Scope

The proposed framework has demonstrated superiority over existing techniques, making it a valuable tool for assisting doctors and reducing the burden on healthcare systems. Overall, the proposed framework has the potential to be a valuable asset in the field of medical diagnosis and could help to improve patient outcomes. Indeed, it is essential to acknowledge that the current evaluation of the models has primarily prioritized accuracy improvement without explicitly considering time and space complexity. This area could be addressed in future research by treating the problem as a multi-objective task that considers the accuracy, F2-score, inference time, and AUC-ROC score as different cost functions during optimization. Different explainable AI tools can be utilized to demonstrate the percentage of nodules correctly classified, which is beyond the scope of this paper. It will be interesting to check the performance of the AI model on two different image modality datasets for the same disease. The proposed framework could also be extended to include quantum machine learning techniques, which have shown promise in other fields. Furthermore, it may be worthwhile to explore the potential of neuromorphic spiking neural networks as an alternative to classical machine learning classifiers in this context, as they have shown promise in achieving high levels of accuracy while consuming relatively low amounts of power and computational resources.

## Figures and Tables

**Table 1:** Performance metrics, TOPSIS scores, and models ranking based on feature extractors and classifiers for ultrasound dataset (5-fold cross validation).

Model	Feature Extraction	Classifier	Accuracy	F2-Score	AUC-ROC	TOPSIS Score	Rank
Model1	DeiT	LR	0.8427	0.8023	0.8929	0.5016	9
Model2		NB	0.7138	0.7036	0.8460	0.2536	13
Model3		SVC	0.9091	0.9069	0.9590	0.8067	6
Model4		KNN	0.7797	0.7302	0.8613	0.3233	12
Model5		RF	0.9685	0.9707	0.9884	0.9716	3
Model6	Swin Transformer	LR	0.8899	0.8565	0.9283	0.6622	7
Model7		NB	0.7028	0.6901	0.7684	0.0807	15
Model8		SVC	0.9231	0.9318	0.9640	0.8621	4
Model9		KNN	0.8444	0.8432	0.9014	0.5878	8
Model10		RF	0.9790	0.9833	0.9885	1.0000	1
Model11	Mixer-MLP	LR	0.8252	0.7844	0.9019	0.4898	10
Model12		NB	0.7028	0.6537	0.7993	0.0903	14
Model13		SVC	0.9126	0.9170	0.9628	0.8319	5
Model14		KNN	0.7885	0.7555	0.8728	0.3853	11
Model15		RF	0.9455	0.9819	0.9884	0.9968	2

**Table 2:** Performance metrics, TOPSIS scores, and models ranking based on feature extractors and classifiers for histopathological dataset (5-fold cross validation).

Model	Feature Extraction	Classifier	Accuracy	F2-Score	AUC-ROC	TOPSIS Score	Rank
Model1	DeiT	LR	0.6541	0.6142	0.7075	0.0485	15
Model2		NB	0.6271	0.6083	0.7292	0.0754	12
Model3		SVC	0.7876	0.6874	0.8878	0.4792	7
Model4		KNN	0.8076	0.7219	0.908	0.5891	6
Model5		RF	0.8712	0.8095	0.9414	0.8764	2
Model6	Swin Transformer	LR	0.6376	0.6217	0.6955	0.0656	13
Model7		NB	0.6188	0.6023	0.7252	0.0633	14
Model8		SVC	0.8135	0.7402	0.9084	0.6413	5
Model9		KNN	0.8624	0.7983	0.937	0.8384	3
Model10		RF	0.8835	0.8461	0.9463	1	1
Model11	Mixer-MLP	LR	0.66	0.6513	0.7249	0.1788	11
Model12		NB	0.6594	0.6232	0.8443	0.2964	10
Model13		SVC	0.7889	0.649	0.8961	0.407	9
Model14		KNN	0.7859	0.6641	0.901	0.4417	8
Model15		RF	0.8541	0.7745	0.9296	0.7595	4

**Table 3:** Weights calculation using MEREC method for the models based on feature extraction and classification techniques.

Criteria	Accuracy	F2-Score	AUC-ROC
Weights1 (Ultrasound Dataset)	0.31123	0.4062	0.2826
Weights2 (Histopathological Dataset)	0.3694	0.2601	0.3704

**Table 4:** Performance metrics, TOPSIS scores, and models ranking based on different dimentionality reduction techniques for ultrasound dataset (5-fold cross validation).

Model	Feature Reduction	Accuracy	F2-Score	AUC-ROC	TOPSIS Scores	Rank
Model10	PCA	0.9790	0.9832	0.9885	0.9679	1
Model16	SVD	0.9720	0.9720	0.9910	0.9465	3
Model17	FAST ICA	0.9825	0.9930	0.9815	0.9270	4
Model18	ISOMAP	0.9825	0.9930	0.9841	0.9466	2
Model19	LLE	0.9335	0.9292	0.9656	0.7418	5
Model20	UMAP	0.8147	0.7015	0.9075	0.0000	6

**Table 5:** Performance metrics, TOPSIS scores, and models ranking based on different dimensionality reduction techniques for histopathological dataset (5-fold cross validation).

Model	Feature Reduction	Accuracy	F2-Score	AUC-ROC	TOPSIS Scores	Rank
Model10	PCA	0.8835	0.8461	0.9463	0.8871	1
Model16	SVD	0.8771	0.8279	0.9443	0.7124	3
Model17	FAST ICA	0.8412	0.7871	0.9030	0.3368	6
Model18	ISOMAP	0.8188	0.8283	0.8971	0.5819	4
Model19	LLE	0.8600	0.8460	0.9245	0.8291	2
Model20	UMAP	0.7988	0.8568	0.8320	0.5377	5

**Table 6:** Weights calculation using MEREC method for the models based on feature reduction techniques.

Criteria	Accuracy	F2-Score	AUC-ROC
Weights3 (Ultrasound Dataset)	0.2963	0.5711	0.1326
Weights4 (Histopathological Dataset)	0.2868	0.2786	0.4346

**Table 7:** Optimal model selection and performance parameters evaluation based on weighted average ensemble and feature selection using FOX-optimization algorithm for ultrasound dataset.

Model	Strategy Used	Accuracy	F2-Score	AUC-ROC	TOPSIS Score	Rank
Model21	PCA + FOX optimization based	0.9913	0.9882	0.9913	1.0000	1
	Feature selection + Random Forest					
Model22	PCA + ISOMAP Weighted Average	0.9825	0.9728	0.9880	0.0000	3
	using FOX-optimization + Random Forest					
Model23	ISOMAP + FOX-optimization based	0.9843	0.9818	0.9888	0.4736	2
	Feature selection + Random Forest					

**Table 8:** Optimal model selection and performance parameters evaluation based on weighted average ensemble and feature selection using FOX optimization algorithm for histopathological dataset.

Model	Strategy Used	Accuracy	F2-Score	AUC-ROC	TOPSIS Score	Rank
Model21	PCA + FOX optimization based	0.9065	0.9201	0.9548	1.0000	1
	Feature selection + Random Forest					
Model22	PCA + LLE Weighted Average	0.8924	0.9092	0.9464	0.5508	2
	using FOX-optimization + Random Forest					
Model23	LLE + FOX optimization based	0.8812	0.8965	0.9259	0.0000	3
	Feature selection + Random Forest					

**Table 9:** Weights calculation using MEREC method for the models based on FOX optimization feature selection and weighted ensemble techniques.

Criteria	Accuracy	F2-Score	AUC-ROC
Weights5 (Ultrasound Dataset)	0.2732	0.6216	0.1052
Weights6 (Histopathological Dataset)	0.2000	0.3356	0.4644

**Table 10:** Comparison with state-of-the-art techniques (ultrasound dataset).

Method	Accuracy	Recall	Specificity
Naguyen et al. [12]	0.9088	0.9493	0.6374
Naguyen et al. [13]	0.9205	0.9607	0.6569
Proposed Model	0.9913	0.9861	0.9965

**Table 11:** Comparison with state-of-the-art techniques (histopathological dataset).

Method	Accuracy	AUC-ROC
Bohland et al. [25] Deep Learning	0.891	0.921
Bohland et al. [25] Feature Extraction Based	0.897	0.93
Proposed Model	0.9065	0.9548

## Data Availability

The histopathology Tharun and Thompson dataset is provided on request by Dr. Lars Tharun and Dr. Lester Thompson. The source of the dataset is https://pubmed.ncbi.nlm.nih.gov/29508145 (accessed on 25 April 2022) with PubMed ID: 29508145.
